# A simple framework for a complex problem? Predicting wildlife–vehicle collisions

**DOI:** 10.1002/ece3.2306

**Published:** 2016-08-18

**Authors:** Casey Visintin, Rodney van der Ree, Michael A. McCarthy

**Affiliations:** ^1^ Quantitative and Applied Ecology GroupSchool of BioSciencesUniversity of MelbourneParkvilleVic.3010Australia; ^2^ Australian Research Centre for Urban EcologyRoyal Botanic Gardens Victoria and School of BioSciencesUniversity of MelbourneParkvilleVic.3010Australia

**Keywords:** Animal, co‐occurrence, kangaroo, risk, road ecology, roadkill, spatial, species distribution model, speed limit, traffic volume

## Abstract

Collisions of vehicles with wildlife kill and injure animals and are also a risk to vehicle occupants, but preventing these collisions is challenging. Surveys to identify problem areas are expensive and logistically difficult. Computer modeling has identified correlates of collisions, yet these can be difficult for managers to interpret in a way that will help them reduce collision risk. We introduce a novel method to predict collision risk by modeling hazard (presence and movement of vehicles) and exposure (animal presence) across geographic space. To estimate the hazard, we predict relative traffic volume and speed along road segments across southeastern Australia using regression models based on human demographic variables. We model exposure by predicting suitable habitat for our case study species (Eastern Grey Kangaroo *Macropus giganteus*) based on existing fauna survey records and geographic and climatic variables. Records of reported kangaroo–vehicle collisions are used to investigate how these factors collectively contribute to collision risk. The species occurrence (exposure) model generated plausible predictions across the study area, reducing the null deviance by 30.4%. The vehicle (hazard) models explained 54.7% variance in the traffic volume data and 58.7% in the traffic speed data. Using these as predictors of collision risk explained 23.7% of the deviance in incidence of collisions. Discrimination ability of the model was good when predicting to an independent dataset. The research demonstrates that collision risks can be modeled across geographic space with a conceptual analytical framework using existing sources of data, reducing the need for expensive or time‐consuming field data collection. The framework is novel because it disentangles natural and anthropogenic effects on the likelihood of wildlife–vehicle collisions by representing hazard and exposure with separate, tunable submodels.

## Introduction

Roads have well‐documented negative ecological impacts (Forman and Alexander 1998; Spellerberg 1998; van der Ree et al. 2015), including effects on terrestrial fauna. Road construction and use fragments and destroys habitat, causes pollution (e.g., noise, light and chemical runoff), and kills and injures animals. Perhaps the most visible impact is direct mortality through wildlife–vehicle collisions (WVC) – billions of fauna are killed annually around the world (Seiler and Helldin 2006). Such an issue has prompted many road management authorities to routinely collect animal carcasses struck and killed by moving vehicles to reduce visual impacts for road travelers (Huijser et al. 2007) and avoid secondary collisions with scavenging wildlife species. In addition, many governments around the world incur significant costs installing wildlife‐proof fencing and under‐ and overpasses to reduce the rate of WVC and improve landscape connectivity (van der Ree et al. 2015).

Worldwide, the frequency, magnitude, and distribution of WVC have been widely studied. Many such studies relate rate of collisions to environmental conditions, anthropogenic variables, and animal biology, behavior, and characteristics. While many studies have used statistical modeling to determine hot spots for WVC, most are limited to a single stretch of road or small collections of roads (Gundersen and Andreassen 1998; Clevenger and Wierzchowski 2001; Clevenger et al. 2003; Ramp et al. 2005; Ramp and Ben‐Ami 2006; Gomes et al. 2008; Hurley et al. 2009; Langen et al. 2009; Roger and Ramp 2009; Hothorn et al. 2012; Markolt et al. 2012; Santos et al. 2013; Seo et al. 2015). These models perform relatively well at local scales, and some have been employed to communicate areas of high risk to road managers, but many cannot extrapolate to other sections of road or entire networks. We extend this work by developing and testing a framework that may be applied at much larger scales, consistent with the boundaries of road authority jurisdictions (i.e., state/provincial). Clevenger et al. (2015) assert that the variability of significant predictors among regions and geographic scales highlights a critical need for a useful broad‐scale conceptual framework to analyze/predict WVC.

Managers often have limited time and budgets to survey WVC across large areas or road networks. Many existing studies utilize data collected at fine spatial scales to model WVC, which is only feasible for limited areas because it is prohibitively expensive to collect data at the required regional scale. Methods to incorporate publicly accessible, existing sources of data or model data‐deficient parameters are useful to reduce costs. For example, remotely sensed data are useful to determine environmental influences, while GIS‐based census data may be used to characterize road conditions. Our framework is a desktop‐based exercise for managers to determine risk across a large network, with the ability to fine‐tune it, based on available data. Moreover, it can be adapted with additional or modified data without changing the underlying methodology.

When modeling is used to determine hot spots for WVC, predictions are made using single models that combine both environmental and anthropogenic variables (Malo et al. 2004; Ramp et al. 2005; Gomes et al. 2008; Roger and Ramp 2009; Hothorn et al. 2012; Barthelmess 2014; Meisingset et al. 2014; Snow et al. 2014). These studies are valuable for managers to identify locations for mitigation or further study; however, they are often limited in their ability to extrapolate beyond the study area and do not clearly indicate potential confounding effects or suggest what mitigation to use (e.g., on the road environment or on the species). For example, vegetation on the road verge is commonly used to model collisions and has been shown to be an effective predictor in previous work. But as a single covariate among others in a regression model, it is difficult to determine whether vegetation is related to collisions based on its affect on visibility for drivers or its attraction for animals. We extend the utility of this research by disentangling the effects of human activity and wildlife behavior, which has not been achieved before. We aim to improve the accessibility of collision analysis and predictions by relating risk to two components, exposure and hazard. This hierarchical classification and structure of the predictors enables a more straightforward calibration and interpretation of the models – a useful feature for managers – without compromising predictive performance.

## Materials and Methods

### Conceptual model framework

Risk (as a rate) of animal collisions can be expressed as a function of exposure and hazard:

(1) $$R_{i}=a\cdot E_{i}\cdot H_{i}$$

where *R*_*i*_ is the risk, *E*_*i*_ is the exposure, *H*_*i*_ is the hazard, *a* is a constant of proportionality, and *i* represents a modeling unit (e.g., site, road, road segment, etc.). This equation is multiplicative and to enable linear analysis, we logarithmically transform the variables:

(2) $$\text{ln}\left(R_{i}\right)=\text{ln}\left(a\right)+\text{ln}\left(E_{i}\right)+\text{ln}\left(H_{i}\right)$$

This suggests risk is perfectly related to both exposure and hazard, and we allow modified responses by expressing the constant of proportionality as an intercept and introducing regression coefficients such that risk is modeled as:

(3) $$\text{ln}\left(R_{i}\right)=\beta_{0}+\beta_{1}\text{ln}\left(E_{i}\right)+\beta_{2}\text{ln}\left(H_{i}\right)$$

‐or equivalently as‐

(4) $$R_{i}=e^{\beta_{0}+\beta_{1}\text{ln}\left(E_{i}\right)+\beta_{2}\text{ln}\left(H_{i}\right)}$$

Here, *β*_1_ and *β*_2_ indicate the relative influence of each predictor on risk. This makes inferences on the model fit more tractable and clearly identifies areas for management focus (e.g., the exposure or the hazard). If risk is exactly related to exposure and hazard as in eq. (2), then both regression coefficients *β*_1_ and *β*_2_ will equal one.

For this study, we represent exposure with animal presence, and hazard with both traffic volume and speed as neither one in isolation would be a realistic threat. We envisage risk as being measured by the rate of collisions. Using this configuration, the rate of collisions (*C*_*i*_) is modeled as a function of three predictors of (1) species occurrence, (2) traffic volume, and (3) traffic speed:

(5) $$C_{i}=e^{\beta_{0}+\beta_{1}\text{ln}\left(O_{i}\right)+\beta_{2}\text{ln}\left(V_{i}\right)+\beta_{3}\text{ln}\left(S_{i}\right)}$$

where *O*_*i*_ is species occurrence, *V*_*i*_ is traffic volume, *S*_*i*_ is traffic speed, in a given place *i*.

While we consider risk as being measured by the rate of collisions (e.g., collisions per month), our data consist of binary events (observations of individual collisions and no collisions, coded as ones or zeroes, respectively). If collisions are treated as events in a Poisson encounter model, then the probability of no collisions occurring equals $e^{-C_{i}}$ Let *Y*_*i*_ = 1 indicate a collision occurred at site *i*, and *Y*_*i*_ = 0 indicate no collision, with *p*_*i*_ = Pr(*Y*_*i*_ = 1). The probability of no collision occurring is equal to one minus the probability of a collision, so it is defined by:

(6) $$\text{ln}\left(1-p_{i}\right)=-e^{\beta_{0}+\beta_{1}\text{ln}\left(O_{i}\right)+\beta_{2}\text{ln}\left(V_{i}\right)+\beta_{3}\text{ln}\left(S_{i}\right)}$$

‐or equivalently as‐

(7) $$\text{ln}\left(-\text{ln}\left(1-p_{i}\right)\right)=\beta_{0}+\beta_{1}\text{ln}\left(O_{i}\right)+\beta_{2}\text{ln}\left(V_{i}\right)+\beta_{3}\text{ln}\left(S_{i}\right)$$

The left‐hand side of eq. (7) is the complementary log–log link function, which has similar properties to the more common logit function of logistic regression. However, we use the complementary log–log function here, because it relates more clearly to the rate of collisions (eq. (5)), and to our conceptual risk model (eq. (1)). Thus,

(8) $$\text{cloglog}\left(p_{i}\right)=\beta_{0}+\beta_{1}\text{ln}\left(O_{i}\right)+\beta_{2}\text{ln}\left(V_{i}\right)+\beta_{3}\text{ln}\left(S_{i}\right)$$

Information on species occurrence, traffic volume, and traffic speed is unlikely to be available for every place *i* and we propose that this can be modeled. Several transportation modeling methods exist for estimating traffic patterns: generalized linear modeling (Seaver et al. 2000; Zhao and Chung 2001), neural network analysis (Duddu and Pulugurtha 2013), empirical Bayes estimation (Yang and Davis 2002), universal kriging (Eom et al. 2006; Selby and Kockelman 2013), and support vector machines (Castro‐Neto et al. 2009). Modeling approaches to estimate species occurrence include correlative (Guisan and Thuiller 2005; Elith and Leathwick 2009) and mechanistic (Kearney and Porter 2009) species distribution modeling (SDM) and extensions of population viability analysis (PVA) (Gilpin and Soule 1986; Shaffer 1990).

**Figure 1:**
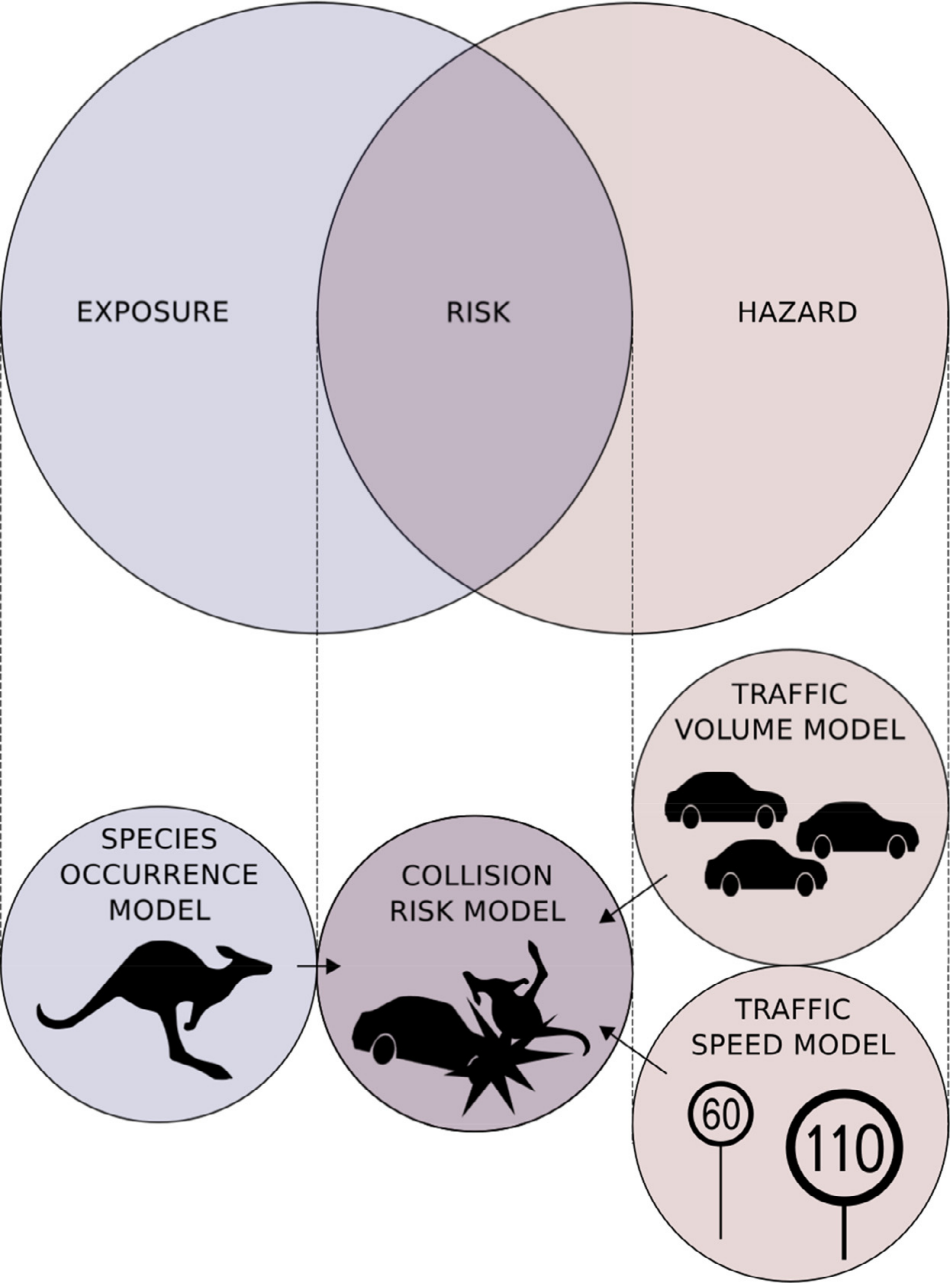
Diagram of modeling framework. Three submodels are used to generate covariates used in the collision model per the “risk equals exposure multiplied by hazard” analytical framework.

We apply our framework to study vehicle collisions with kangaroos in Australia using SDM to estimate kangaroo occurrence and linear regression to estimate traffic volume and speed (Fig. 1).

### Study area and species

We selected the State of Victoria in southeast Australia as a study area as its geographic diversity across its area of 227,819 square kilometers (Australian Bureau of Statistics, 2011) provides a good platform to illustrate the framework. Our study combines all sealed roads within the state (approx. 150,000 km) and predicts collision risk across six motorway class types. To organize our spatial data, we overlaid a spatial grid of one square kilometer resolution on the study area. To produce modeling units for the collision model, we further segmented the roads by intersecting all roads and the spatial grid. The open‐source software package “R” (R Development Core Team, 2004) was used to perform all spatial and statistical analyses.

We used the native species Eastern Grey Kangaroo (*Macropus giganteus*, Shaw, hereafter referred to as “grey kangaroo”) as the case study species. In Victoria, the grey kangaroo is the most abundant of the macropod family and frequently involved in WVC. Between 2005 and 2013, over 600 incidents were reported to the Victorian Police and documented in the VicRoads crashstats database (VicRoads, 2014). Actual incident rates, however, are much higher as the largest Victorian wildlife organization, Wildlife Victoria, received over 5000 reports of grey kangaroo–vehicle collisions over the same period (Wildlife Victoria, 2014). Grey kangaroos are the second largest native terrestrial mammal in Australia and share many similar characteristics and management issues with ungulates found in North America and Europe (Croft 2004; Coulson and Eldridge 2010).

### Species occurrence submodel

We downloaded survey records of grey kangaroos from the Victorian Biodiversity Atlas (VBA) occurring in the period 2000–2014 and spatial accuracy within 500 m (DELWP, 2015). The presences were both systematic targeted surveys and incidental sightings from data maintained by the Arthur Rylah Institute, a division of the Victorian Department of Environment, Land, Water and Planning. Kangaroos are generalists and widely distributed; it was therefore assumed that although abundance may fluctuate based on drought conditions, distribution would not change significantly from the year 2000. We used data from this period because it improved the sample size for occurrence modeling. We derived background data by randomly sampling 10,000 points across the entire study area. After sampling values from the covariate rasters and eliminating null values, the final dataset included 901 species presence and 9957 background observations.

**Figure 2:**
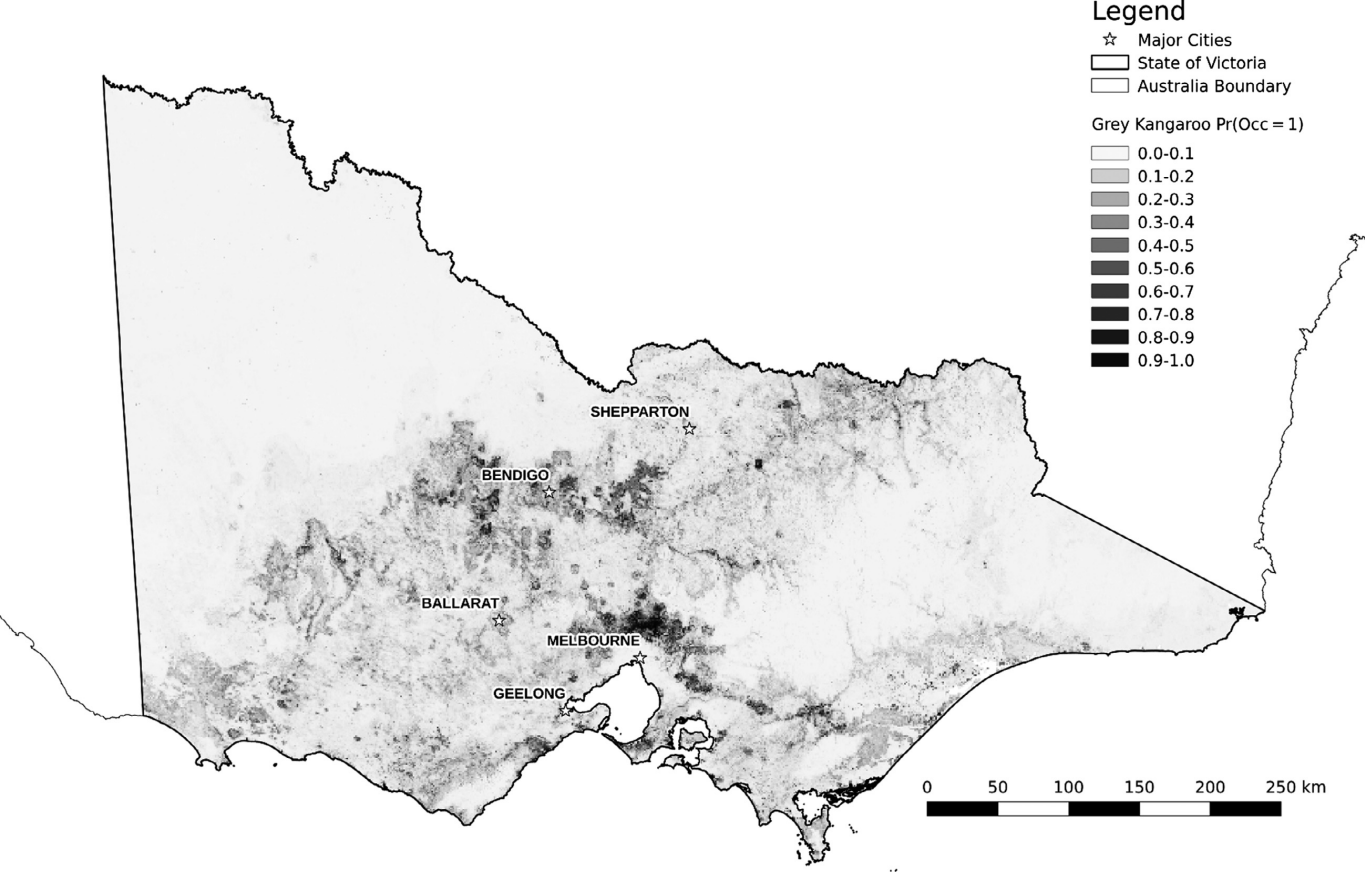
Predicted relative likelihood of grey kangaroo presence in study area. Darker shades indicate higher relative probabilities of occurrence (mean: 0.057; range: 0.002–0.986).

To estimate occurrence of grey kangaroos, we used boosted regression trees (BRT) (Friedman 2002). BRT modeling offers advantages of handling different types of predictor variables, accommodating missing data and outliers, fitting complex nonlinear relationships, and incorporating interaction effects between predictors (Elith et al. 2008). We selected a tree complexity of five (limit on number of terminal nodes per tree used to include potential interactions) and a learning rate of .005 (contribution of each tree to the model). Classification methods have an established use in studies of species distributions (Walker 1990; Skidmore et al. 1996); however, our framework is not limited to any particular modeling method. We selected seven predictors (Table 1) based on the biology and behavior of grey kangaroos. All of the species occurrence submodel predictor variables were below a pairwise correlation threshold of 0.75 to reduce potential effects of multicollinearity. We predicted relative likelihood of grey kangaroo occurrence from the model fit at a one square kilometer resolution across Victoria (Fig. 2).

**Table 1:** Variables used in statistical models

Model	Variable	Definition
Species occurrence	KANG	Presences and psuedo‐absences of Eastern Grey Kangaroos
	ELEV	Elevation of terrain in meters above sea level
	GREEN	Remote‐sensed mean seasonal change in greenness (2003–2013) in vegetation
	LIGHT	Remote‐sensed relative artificial light intensity
	MNTEMPWQ	Mean temperature of wettest quarter in °C
	PRECDM	Precipitation of driest month in millimeters
	SLOPE	Slope of terrain in decimal percent rise
	TREEDENS	Tree canopy coverage within 1 square kilometer in decimal percentage
Traffic volume	AADT	Average annual daily traffic counts per road segment
	KMTODEV	Distance in kilometers to urban land use
	KMTOHWY	Distance in kilometers to major road segments (freeways and highways)
	POPDENS	2011 Population divided by area in square kilometers
	RDCLASS	Road class (“freeway,” “highway,” “arterial,” “subarterial,” “collector,” or “local”) – proximal measure of intensity
	RDDENS	Total length in kilometers of road segments within 1 square kilometer
Traffic speed	SPEEDLMT	Posted speed limit per road segment
	RDCLASS	Road class (see above)
	RDDENS	Total length in kilometers of road segments within 1 square kilometer
Collision	COLL	Presences and psuedo‐absences of grey kangaroo–vehicle collisions
	EGK	Predicted relative likelihood of kangaroo presence
	TVOL	Predicted traffic volume (number of vehicles per day) per road segment
	TSPD	Predicted posted traffic speed (kilometers per hour) per road segment

### Traffic volume and speed submodels

**Figure 3:**
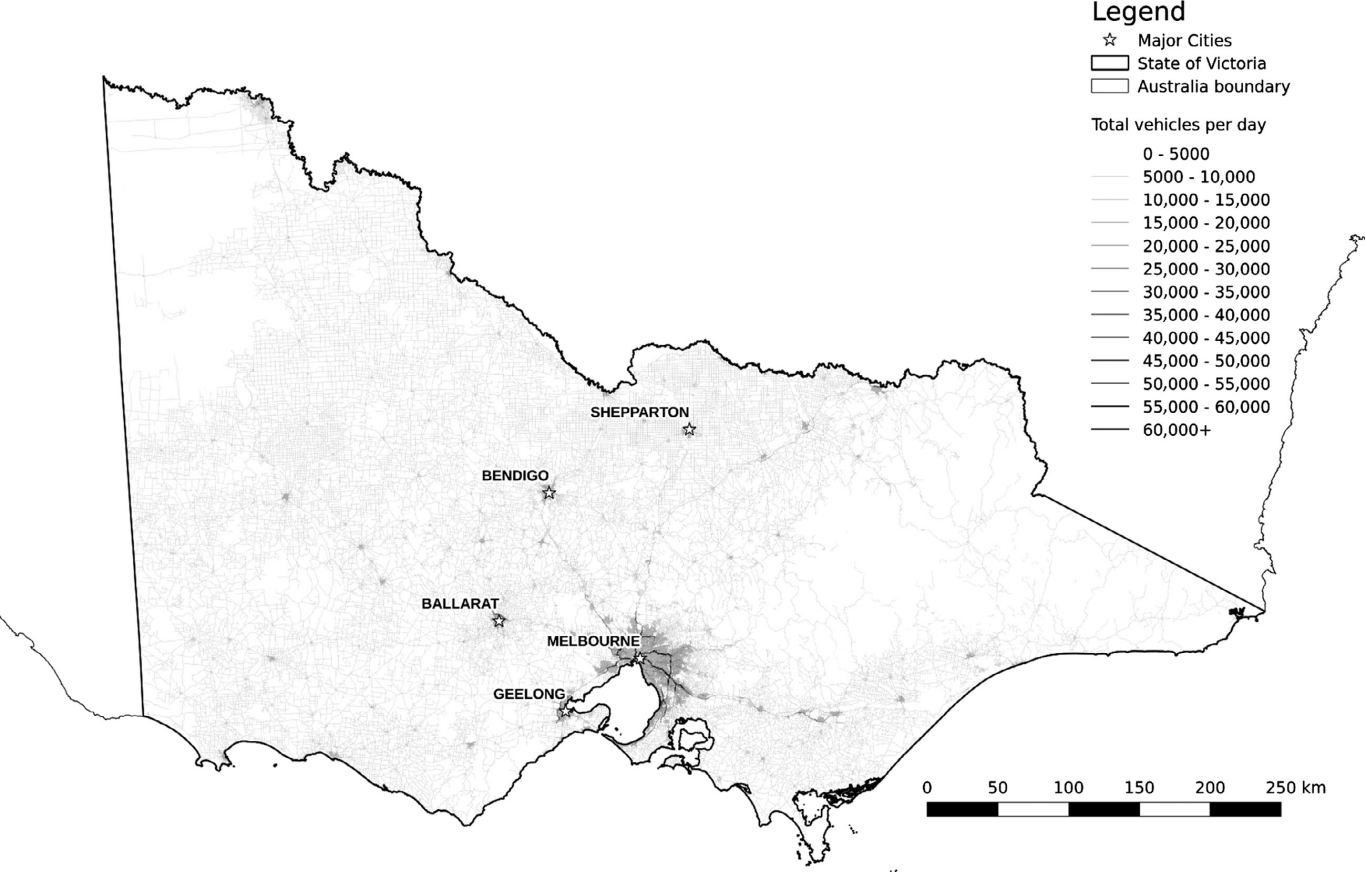
Predicted relative traffic volume in number of vehicles per day per road segment in study area. Darker shades indicate higher predicted traffic volumes (mean: 4481; range: 274–60850).

Average annual daily traffic (AADT) represents the sum of traffic traveling in both directions which pass a roadside observation point during a full year divided by 365 days for a given road segment. AADT volume is usually only available for major road segments, and we did not have data for most local, collector, and subarterial roads under municipal district control. We predicted volume estimates for all road segments in the study area with random forests regression (Breiman 2001). The dependent variable was 2013 AADT recorded by VicRoads on 3174 road segments. We included seven predictor variables (Table 1) that related to processes in traditional four‐step traffic demand modeling (trip generation, trip distribution, mode choice, and route assignment). All of the traffic model predictor variables were below a pairwise correlation threshold of 0.7 to reduce potential effects of multicollinearity. The traffic volume submodel used the log‐link function on the dependent variable (Table 2) due to the approximate log‐normal distribution of AADT. We predicted AADT to all road segments using the model fit (Fig. 3).

**Table 2:** Statistical models used in framework

Model type	Model	Reduction in error (%)	ROC (AUC)
Species occurrence	Pr(KANG = 1) ≈ logit^‐1^(*β*_0_ + *β*_1_ELEV + *β*_2_GREEN + *β*_3_LIGHT + *β*_4_MNTEMPWQ + *β*_5_PRECDM + *β*_6_SLOPE + *β*_7_TREEDENS)	30.4	0.88
Traffic volume	ln (AADT) ≈ *β*_0_ + *β*_1_KMTODEV + *β*_2_KMTOHWY + *β*_3_POPDENS + *β*_4_RDDENS + *β*_5_RDCLASS	54.4	–
Traffic speed	SPEEDLMT ≈ *β*_0_ + *β*_1_RDCLASS + *β*_2_RDDENS	58.7	–
Collision	cloglog(Pr(COLL = 1)) ≈ *β*_0_ + *β*_1_ ln (EGK) + *β*_2_ ln (TVOL) + *β*_3_ ln (TSPD)	23.7	0.81
Alternative collision	cloglog(Pr(COLL = 1)) ≈ *β*_0_ + *β*_1_ELEV + *β*_2_GREEN + *β*_3_KMTODEV + *β*_4_KMTOHWY + *β*_5_LIGHT + *β*_6_MNTEMPWQ + *β*_7_POPDENS + *β*_8_PRECDM + *β*_9_RDCLASS + *β*_10_RDDENS + *β*_11_SLOPE + *β*_12_TREEDENS	24.9	0.84

**Figure 4:**
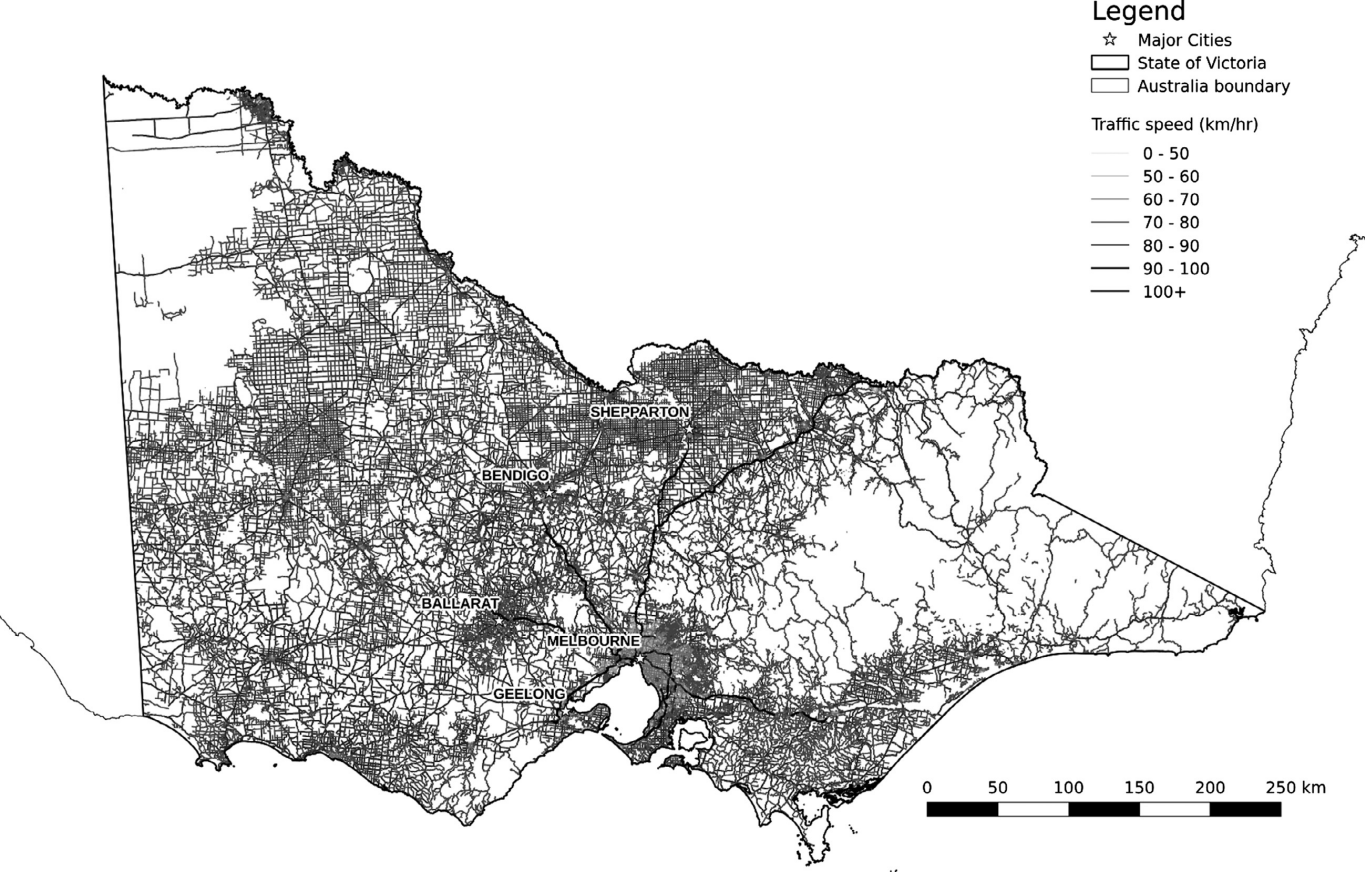
Predicted relative traffic speed in kilometers per hour per road segment in study area. Darker shades indicate higher predicted traffic speeds (mean: 62; range: 42–106).

Traffic speed was modeled and predicted using a similar methodology to the traffic volume model. As with the traffic volume data, we did not have access to municipal records; however, we required speed values for all road segments across Victoria. We obtained 2014 posted speed limit data for all major road segments (*n* = 42,439) and used road density and road class predictors (Table 1) in a random forests regression model (Table 2) to predict speed for all road segments (Fig. 4).

### Collision modeling

To produce a kangaroo–vehicle collision dataset, we obtained records from the Wildlife Victoria database for a 3‐year period between 1 January 2010 and 1 January 2013. The data for collisions were selected from a period where consistent techniques were used to collect and record the data. Pre‐2010 records exist, however, are sparse and more prone to error. We first verified all automatically geocoded coordinates for accuracy using an online latitude/longitude mapping system (Schneider 2015) and excluded all records with a geographical accuracy exceeding 300 m. We selected road segments that were closest to the collision records in space and coded them with ones. To produce background points, we randomly selected approximately twice the number of collision‐coded road segments and coded them with zeros. We combined the collision and background segments to produce a final dataset of 2264 collision and 4489 background segments. Using the same methodology, we developed an additional dataset of road segments for a period of 1 year between 1 January 2013 and 1 January 2014 for model validation (2125 collision and 4212 background records). Each segment contained predicted values for both traffic speed and volume from the previously described submodels. As species occurrence predictions were expressed across a one square kilometer raster grid, we used the midpoint of each road segment for sampling the species occurrence submodel predictions.

**Figure 5:**
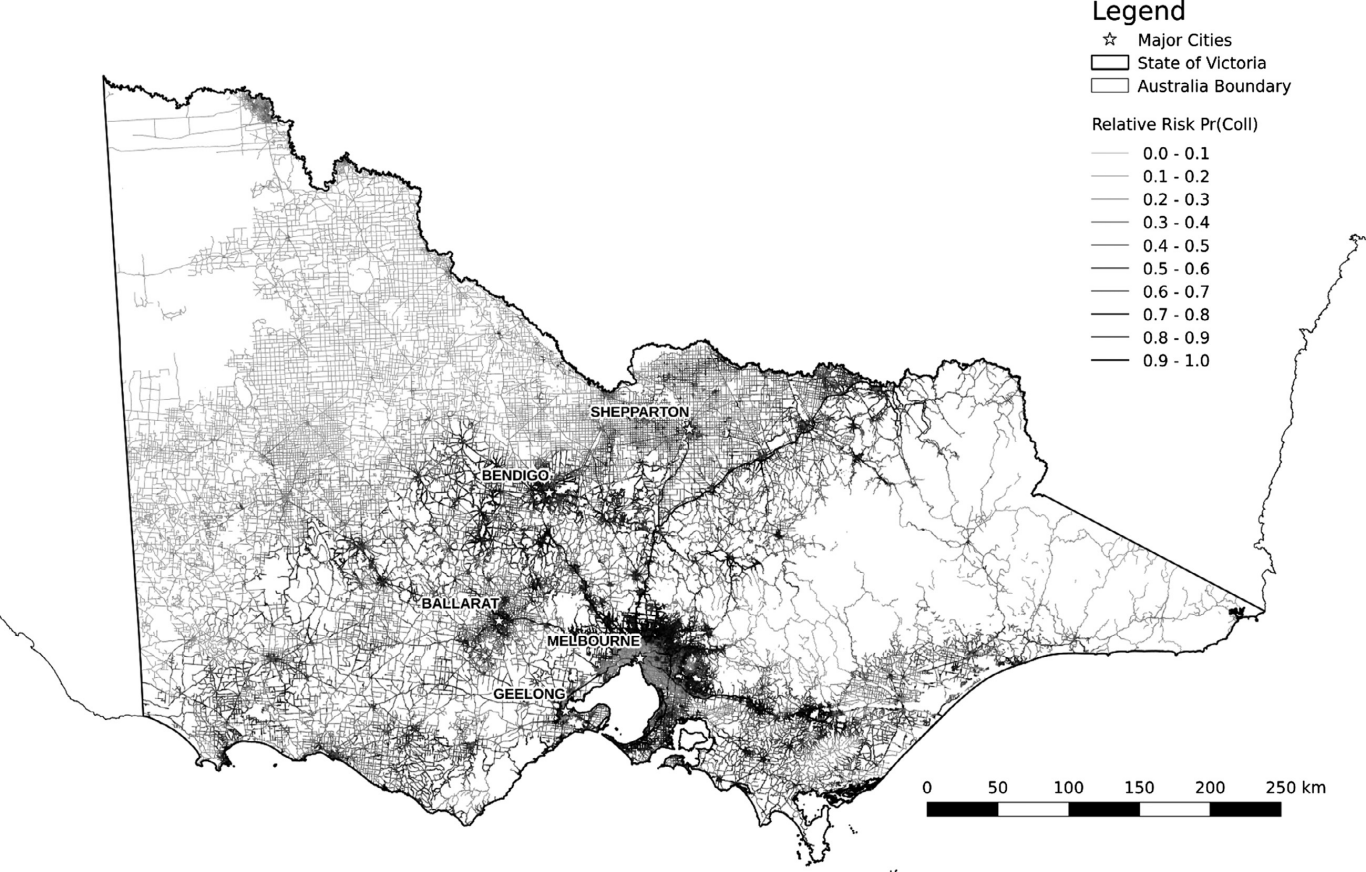
Map of collision risk per road segment. Darker shades indicate higher relative risk of collisions with kangaroos (mean: 0.24; range: 0.01–0.99).

Our collision model fitted predicted values from the submodels to collision or background occurrences at each road segment (Table 2). We used the fitted collision model to predict relative probabilities of collision on all road segments in the study area and, using symbol classification (color and line thickness) in Quantum GIS (QGIS Development Team, 2015), produced a map (Fig. 5).

To determine the effectiveness of partitioning the variables into logical submodels, we also modeled collision risk with a combined set of all variables (Table 2); that is, we analyzed a logistic regression model that related collisions directly to the original variables used to model kangaroo occurrence, traffic volume, and traffic speed. This emulated past work for purposes of comparison. We also compiled a list of variables used in 74 wildlife–vehicle collision modeling papers and verified that nearly all of our predictors were used in at least five or more published studies. The remaining highly used variables were either irrelevant to our scope or difficult to obtain.

## Results

The species occurrence model predicted the relative likelihood of kangaroo presence to vary (0.002 to 0.986) across the study area using 5400 regression trees. The deviance explained by the model was approximately 30.4% (null deviance = 0.572, estimated cross‐validation deviance = 0.398 ± 0.008, cross‐validation AUC = 0.878 ± 0.006; see Table 2). The three most influential variables were artificial light (19.6% contribution to model), elevation (18.4%), and precipitation of the driest month (14.8%). Partial dependence plots demonstrated plausible relationships between the predictors and grey kangaroo presence (see Fig. S1). We extended our model predictions to continental Australia (including areas well beyond Victoria), which aligned well with known grey kangaroo range (see Fig. S2). Approximately fifty percent of the grey kangaroo collision records were within areas where the predicted probability of grey kangaroo occurrence was above 0.2. Collision records in areas of lower predicted occurrence areas may be due to misclassification errors (i.e., misidentification of the species of kangaroo), reporting bias/spatial error in the collision data, or sampling bias in the data used to train the kangaroo occurrence model.

The traffic volume model explained 54.4% of the variation in the AADT data and the traffic speed model explained 58.7% of the variation in the posted speed limit data (Table 2). Each model used the default value of 500 trees to fit the data. All predictor variables used in the traffic models demonstrated plausible relationships to both AADT and speed (see Fig. S3); traffic volume increased with decreased road segment distance to activity centers and major thoroughfares. The most influential variable for traffic volume was road class (33.8% relative contribution to reduction of variance), followed by distance to urban development (19.7%) and distance to freeways and highways (16.4%).

**Figure 6:**
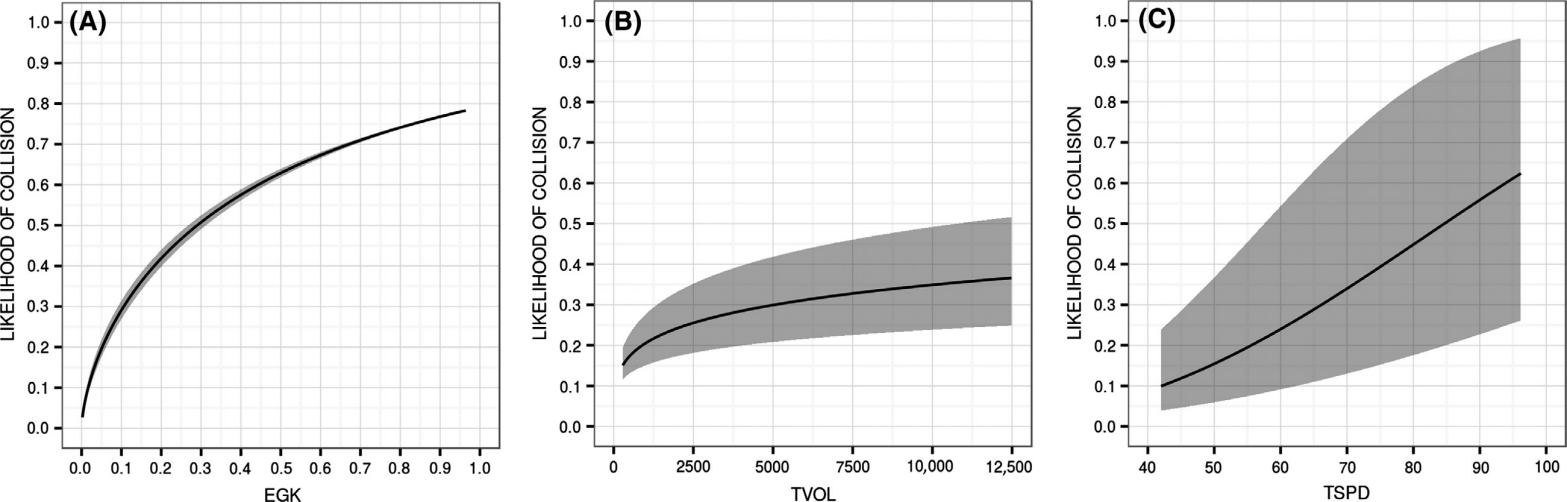
Effects of predictor variables on relative likelihood of collision. EGK is the relative likelihood of kangaroo occurrence (A). TVOL is the predicted daily traffic volume in vehicles per day (B). TSPD is the predicted traffic speeds in kilometers per hour (C). Shaded regions indicate error bounds (95% confidence) on coefficient estimates.

The collision model explained 23.7% of the deviance (Table 2). Using the independent dataset to verify the predictive accuracy of the model resulted in a receiver operating characteristic (ROC) score of 0.81. All three variables were highly significant (table 3) with traffic speed and grey kangaroo occurrence contributing the most to overall reduction in deviance; 27.3% and 72.7%, respectively. The Akaike information criterion (AIC) score – measuring the quality of the model given the data and the parameters – was 6579. All predictor variables demonstrated logical relationships to collision likelihood in the partial dependency plots (Fig. 6). The rapid ascent and gradual leveling off of collision risk to increasing traffic volume suggests a threshold of around 2000 vehicles per day. In very high traffic volume areas, roads may deter animal movement, thereby lessening potential collisions (Seiler 2005; Seiler and Helldin 2006; Gagnon et al. 2007).

**Table 3:** Summary of collision model fit. Coefficients and significance of variables are shown with relative contribution to model fit. Highly significant variables are marked with an asterisk. ANOVA contribution of variables are expressed as decimal percent reduction in deviance

Model type	Variable	Coefficient	SE	*z*‐Value	Pr(>|*z*|)	ANOVA (Contribution)
Collision	Intercept	−12.82	0.6635	−19.33	*<*2e‐16*	–
	EGK	0.6583	0.0206	32.01	*<*2e‐16*	0.7268
	TVOL	0.2715	0.0252	10.77	*<*2e‐16*	0.0005
	TSPD	2.694	0.1308	20.59	*<*2e‐16*	0.2726
Alternative Collision	Intercept	−2.567	0.2642	−9.716	*<*2e‐16*	–
	ELEV	0.003177	0.0002233	14.23	*<*2e‐16*	0.1729
	GREEN	1.405	0.344	4.085	4.42e‐05*	0.0011
	KMTODEV	−0.02784	0.002097	−13.28	*<*2e‐16*	0.2079
	KMTOHWY	0.006001	0.004206	1.427	0.1537	0.0004
	LIGHT	0.004142	0.002046	2.025	0.043	0.0119
	MNTEMPWQ	0.1519	0.01673	9.077	*<*2e‐16*	0.0398
	POPDENS	−0.0006986	0.00005348	−13.06	*<*2e‐16*	0.0922
	PRECDM	0.02573	0.003404	7.559	4.05e‐14*	0.0483
	RDCLASS	−0.4166	0.01467	−28.4	*<*2e‐16*	0.4205
	RDDENS	0.02353	0.008762	2.686	0.0072	0.0038
	SLOPE	0.008252	0.00739	1.117	0.2641	0.0004
	TREEDENS	−0.1761	0.1387	−1.27	0.2041	0.0008

Our alternative collision model (all submodel predictor variables combined in a single model) explained 24.9% of the deviance; <2% more than the four‐model framework (Table 3). The model AIC score was 6496.2. The most influential variables were road class (42.1% relative contribution to reduction of deviance), distance to urban development (20.8%), elevation (17.3%), and population density (9.2%). We used the same independent dataset to verify the predictive accuracy of the alternative model, resulting in an ROC score of 0.84.

## Discussion

This research introduces a new conceptual framework for predicting risk of WVC. It is distinct from other collision models in its treatment of the analytical modeling framework. Where other research has related multiple environmental and anthropogenic variables to collision occurrence in a single statistical model (e.g., Lee et al. 2004; Ramp et al. 2005; Klöcker et al. 2006; Litvaitis and Tash 2008; Gunson et al. 2009; Roger and Ramp 2009) and predicted risk from model fits (e.g., Malo et al. 2004; Sudharsan et al. 2009; Gunson et al. 2011), we separate and develop the predictors in submodels that would most suitably inform management. Each submodel may be independently scrutinized for bias, uncertainty, and spatial autocorrelation and tuned accordingly. In this case study, our approach identifies relationships between, and effects of, species presence and traffic volume/speed on collision risk to grey kangaroos. In a similar manner, Bauduin et al. (2013) developed an index of co‐occurrence to assess collision risk between manatees and recreational watercraft; however, the management implications were not extensively discussed. Particular to our study, road managers and environmental managers may be interested in whether to reduce collisions by focusing on the road environment (e.g., Clevenger and Wierzchowski 2001; Jaeger and Fahrig 2004; Bond and Jones 2008, 2013), traffic conditions, or species (e.g., Huijser and McGowen 2003; Huijser et al. 2006). Both the collision and alternative collision models had similar fits and made similar predictions. However, we argue that cause and effect is easier to interpret when using our proposed framework. For example, the variable of population density (human) is shown to contribute to collisions; however, it is unclear to what degree it is correlated with grey kangaroo occurrence, traffic conditions, or both, in the alternative collision model.

The models used in the study were all correlative and assumed static equilibrium in the environment. Temporal patterns of WVC exist and would be useful to incorporate into the collision model, but the variable of time was not easily integrated into this study. The original collision dataset indicated that the lowest number of incidents occurred in summer (December–February) and the highest were reported in winter (August). The times of highest density of reports were consistent with the crepuscular (most active at dusk and dawn) nature of kangaroo movements (McCullough and McCullough 2000) and peaked at approximately 7:00 and 17:00 h. Comparing the model performance on data accounting for time of day and of year is an area for future research. Other modeling methods that explicitly address interactions between space and time exist, such as multidimensional Poisson process models, and would be useful to incorporate into the framework.

This study demonstrates the usefulness of existing sources of data for scientific research and environmental management. Data provided by non‐governmental organizations, such as Wildlife Victoria, are not only inexpensive to collate, but are also valuable ecological indicators and sources of information, in particular, on species distributions. However, it is cautioned that use of such data should be subject to rigorous quality control and verification. A large amount of entered records were not useful for the scale of this study due to incomplete data or geographical ambiguity. Fauna atlases have the same potential issues. Many of the records in the VBA were subject to geographical bias (e.g., close to roads and towns) and some potential inaccuracies (e.g., misidentification of species or approximate location). Graham et al. (2004) elaborates on the use of such data in scientific studies. Moreover, testing the model with alternative sources of collision reports (e.g., insurance records) would help account for biases present in the data. Although underreporting of collision data has limited effects on model robustness, spatial biases can adversely affect model performance (Snow et al. 2015).

Our statistical modeling methods were chosen to match the data and purpose (as suggested by Wintle et al. 2005; Guillera‐Arroita et al. 2015), and are based on well‐established analyses in the modeling literature. However, our framework is not restricted to the particular models that we built; any appropriate statistical methods may be used for each of the submodels. For example, BRT are a well developed method for SDM (Elith et al. 2008) but may also be suited to predict traffic volume and speed. Further exploration of modeling methods, including those based on machine learning, may result in better calibrated models with less uncertainty and more robust inferences. Moreover, the use of mechanistic models to explain population dynamics (requiring collection of additional information such as age, sex, and size of species involved in WVC) may be beneficial as the integration of PVA into species distribution and collision models can be informative (Tyre et al. 2001; Elith et al. 2010; Polak et al. 2014).

All of the models with binary dependent variables were subject to spatial autocorrelation producing potentially biased standard errors and predictions of grey kangaroo presence and collision risk. Techniques to incorporate autocovariates in the models similar to Crase et al. (2012) might improve the predictive performance but may only be logical for particular species (e.g., ranging or nomadic species). Further, we chose to test the model framework using a binomial dependent variable; however, it can be adapted to analyze other data types such as non‐negative integers (e.g., number of collisions) or categorical information (e.g., groupings of number of collisions). These alternative methods might usefully identify hotspots (i.e., sites with multiple collisions) at smaller scales such as road segments or simulate effects of planned road expansion projects. For example, population effects on target species can be quantified and estimated based on predicting the magnitudes of collision hotspots.

### Applied management implications

To realize the full potential use by managers, a model framework must be conceptually simple, flexible, and adaptable. All of the input data must be accessible, and the framework must allow inferences which are relevant and draw conclusions which are tractable. Our study uses vehicle collisions with grey kangaroos in Australia to demonstrate this analytic framework; however, we envision extending the model to (1) explore and predict risk to other species (e.g., wombats or deer) arising from vehicle collisions by identifying and quantifying probabilities of occurrence using alternative species distribution models and (2) quantify risks to wildlife arising from other anthropogenic threats such as linear infrastructure (e.g., electrocutions), pollution (e.g., entanglements), or introduction of domestic predators (e.g., dog and cat attacks). Managers can use our framework across several disciplines, and it would benefit from additional testing across several spatial and temporal scales to determine its full potential flexibility and generality.

## Conflict of Interest

None declared.

## Data Accessibility

Collision model dataset: archived on Github
R script for collision model: archived on Github

## Supporting information


**Figure S1.** Effects of predictors on relative likelihood of grey kangaroo occurrence.

**Figure S2.** Predicted relative likelihood of grey kangaroo presence across Australia.

**Figure S3.** Effects of predictor variables on traffic volume and speed.
