# Supplementary figures and images for: A simple framework for a complex problem? Predicting wildlife–vehicle collisions
Source: Ecol Evol. 2016 Aug 18;6(17):6409–21. doi: 10.1002/ece3.2306 (PMC5016659; doi:10.1002/ece3.2306)

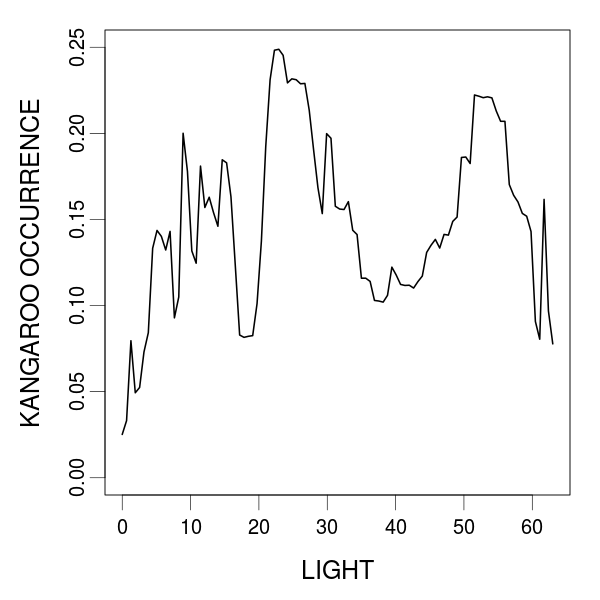

Supplement: Supplementary file 1 — Figure S1. Effects of predictors on relative likelihood of grey kangaroo occurrence. [file ECE3-6-6409-s001.tif]

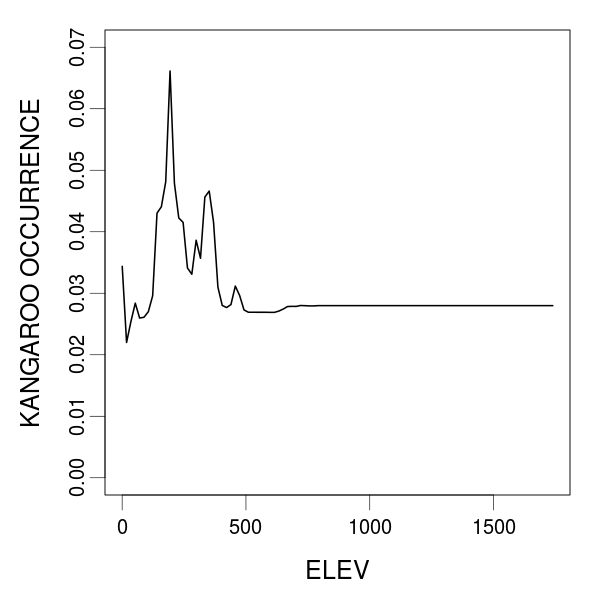

Supplement: Supplementary file 2 [file ECE3-6-6409-s002.tif]

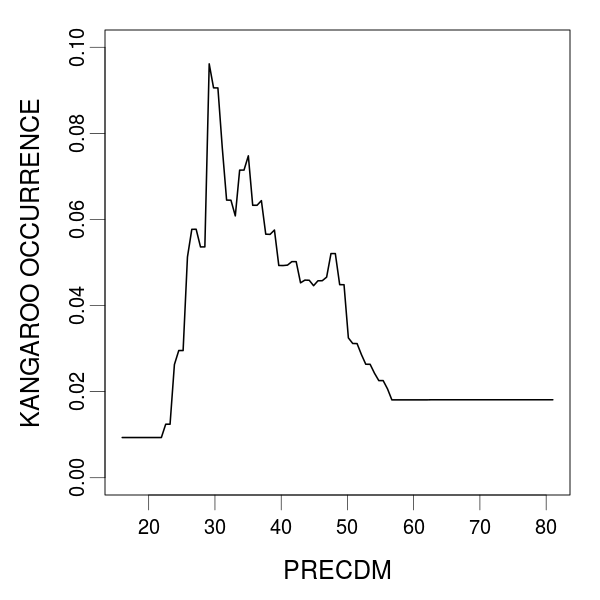

Supplement: Supplementary file 3 [file ECE3-6-6409-s003.tif]

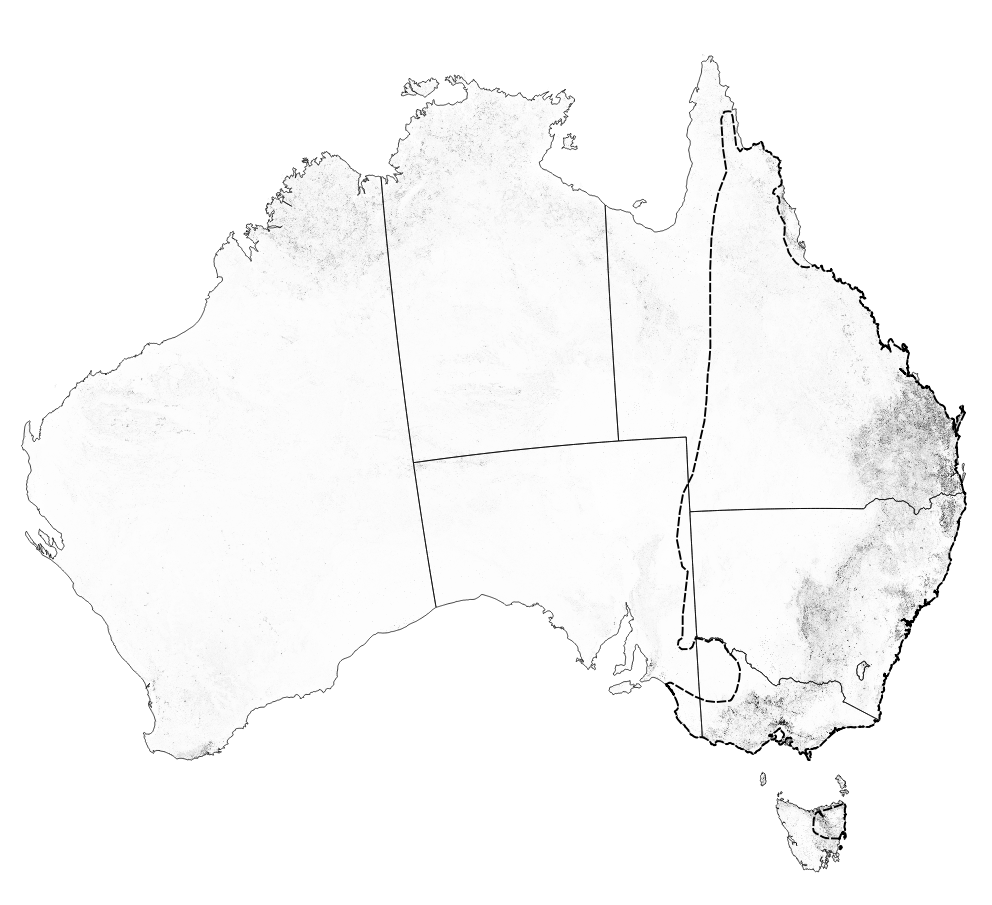

Supplement: Supplementary file 4 — Figure S2. Predicted relative likelihood of grey kangaroo presence across Australia. [file ECE3-6-6409-s004.tif]

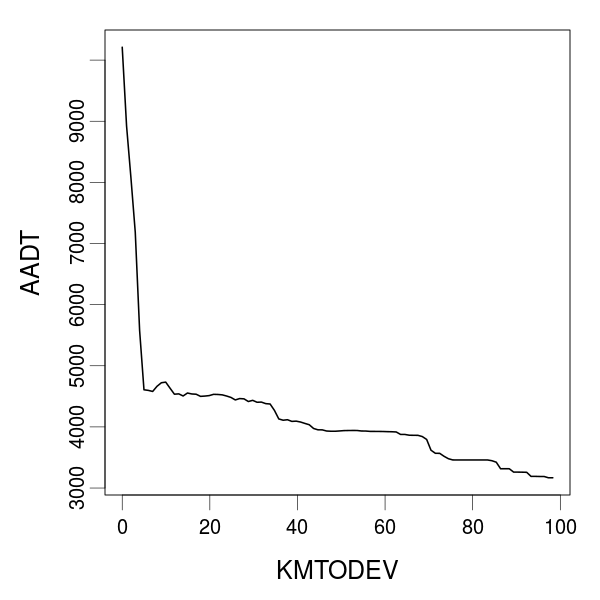

Supplement: Supplementary file 5 — Figure S3. Effects of predictor variables on traffic volume and speed. [file ECE3-6-6409-s005.tif]

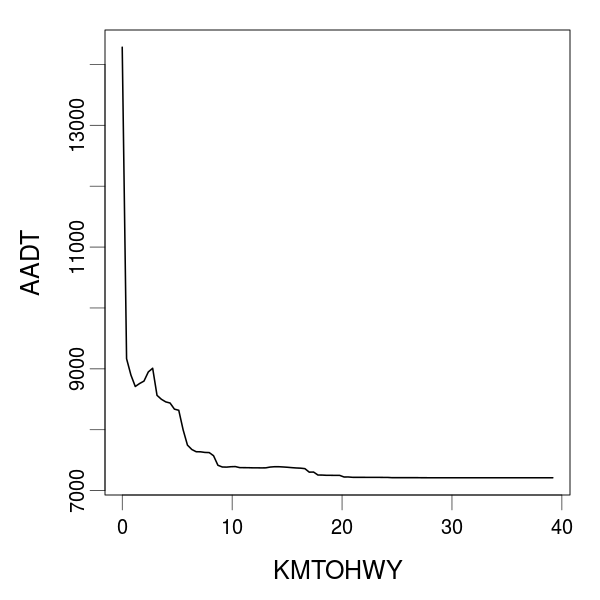

Supplement: Supplementary file 6 [file ECE3-6-6409-s006.tif]

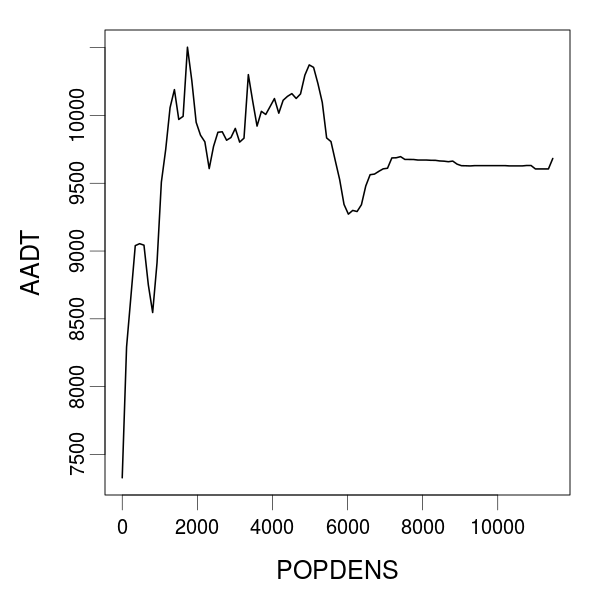

Supplement: Supplementary file 7 [file ECE3-6-6409-s007.tif]

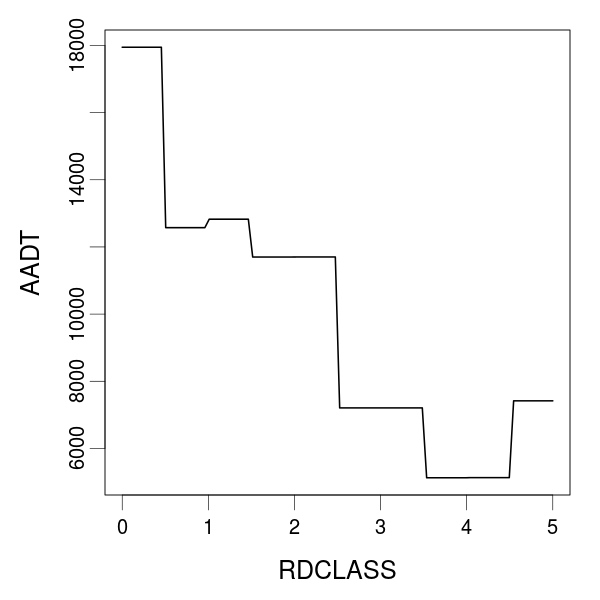

Supplement: Supplementary file 8 [file ECE3-6-6409-s008.tif]

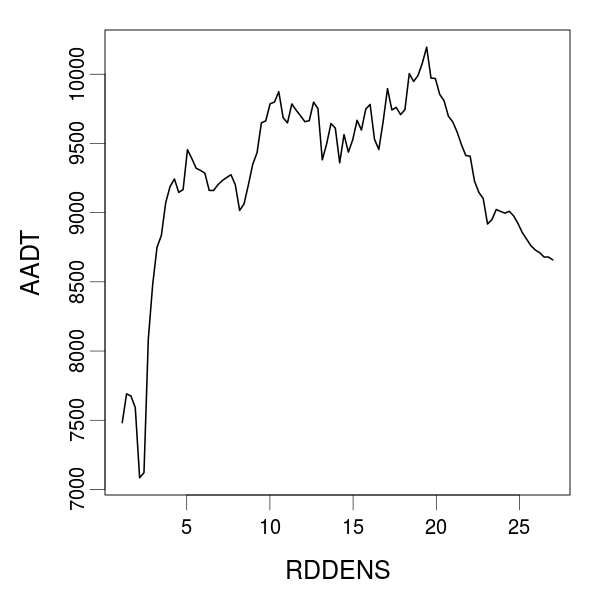

Supplement: Supplementary file 9 [file ECE3-6-6409-s009.tif]

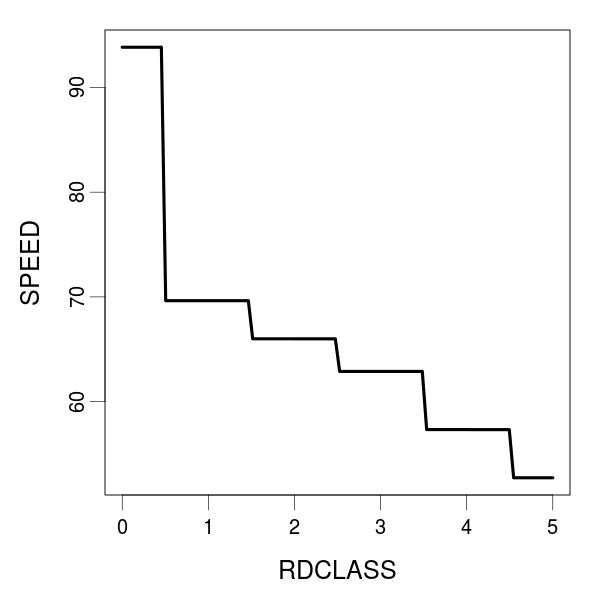

Supplement: Supplementary file 10 [file ECE3-6-6409-s010.tif]

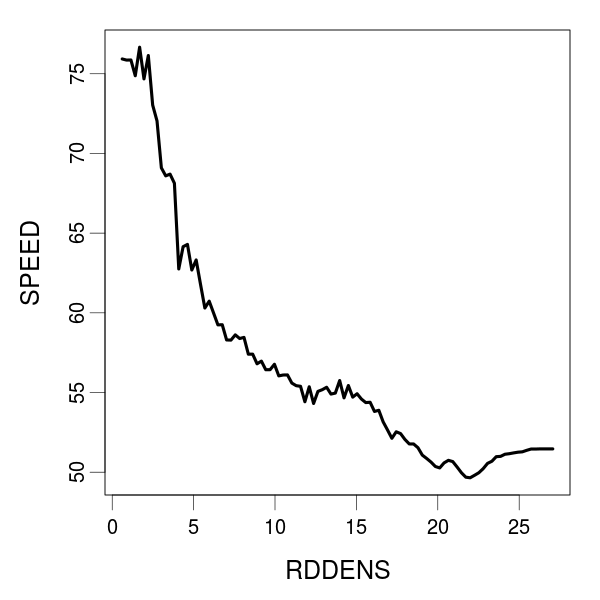

Supplement: Supplementary file 11 [file ECE3-6-6409-s011.tif]
